# Malignant Tumors

**Published:** 1828-03

**Authors:** 


### MALIGNANT TUMORS.

### *Cases of Malignant Tumor affecting different Organs.*

### Case I. *Carcinoma of the Cæcum in a Boy.*

George Wilmot, aged nine years, of a strumous habit, and much emaciated, was admitted into St. George's Hospital, under the care of Dr. Chambers, on the 28th of November, with dropsy of the legs, thighs, and abdomen. Besides the fluid in the belly, several hard immovable tumors were felt, occupying the hypogastric and umbilical regions, and extending upwards into the right hypochondrium. These tumors formed an immovable mass, of an irregular surface, and seemed for the most part attached to the right side of the spine.

The previous history of this case was, that, three years before his admission, he had suffered from an attack of continued fever, after which he remained for a long time very weak and low; that two years ago the belly became distended, and the hard tumors were then first perceived; his legs had swelled only three months before his admission.

He was now placed on a light farinaceous diet, and ordered to take three grains of Calomel every night, a purgative every other morning, and diuretic draughts each day.

Under this treatment the dropsical swellings were nearly removed in the course of ten days. The motions became of a better colour, and the urine ceased to deposit any sediment; but no alteration was observable in the hard tumors, and the tenderness of the parietes of the right side of the abdomen was still distressing. There was, however, neither tension nor fever, but excessive debility.

The Calomel was now discontinued, and half a drachm of Linimentum Hydrargyri was ordered to be rubbed in over the abdomen every night; his bowels were kept open by ordinary means; his pain was alleviated by opiates; and other indications, as they arose, were met by the usual treatment, which it is not necessary to describe at length.

He died on the 19th of December, and the next day his body was examined.

There were no traces of inflammation of the peritoneum. A large, irregularly shaped, whitish mass was observed occupying the hypogastric, right iliac, and umbilical regions, and extending up to the inferior surface of the liver. On cutting into the tumor, it was found that the interior of the cæcum and ascending colon, of which parts it consisted, was throughout in a state of foul ulceration; whilst the tunics of the intestine, as far as they could be made out in the midst of so much disorganization, were enormously thickened and hardened: the thickened and indurated parts had the character of true scirrhus, and the ulcerated surface was studded with processes of malignant growth, and had the common aspect of open cancer. The canal of the intestine was not obstructed, and the diseased surface was smeared with healthy feculent matter. Most of the absorbent glands in the neighbourhood of the tumor were enlarged and indurated, and some of them were in a state of unhealthy suppuration. In many other parts of the intestinal canal, particularly of the ileum, a growth of a scirrhous character was observed amongst the coats of the bowel. Some of the glands in the mesentery were enlarged. There appeared also in the cortical part of each kidney the commencement of a disease similar to that observed in the bowels.

Dr. Chambers remarks, that there are few, if any, instances on record of true cancer occurring at so early an age as that of the patient whose case we have just related,—namely, nine years. It is true that what was called cancer by the older writers is by no means a rare disease in infants and very young children; but modern pathologists well know that this latter affection is a fungoid disease, of a species altogether different from true carcinoma, which is now justly considered a disorder, for the most part, of advanced, or at any rate of adult, age. Some light may, perhaps, be thrown on the early occurrence of cancer in this boy by a reference to the history of his case. It appeared that his first complaint was a continued fever, which occurred three years ago, from the effects of which he never entirely recovered, and to which, in fact, his friends attributed his abdominal disease. Now, when it is recollected that almost all the fevers which prevailed in this district three years ago were attended with considerable inflammation of the mucous membrane lining the cæcum, as well as the ileum, and many of them with ulceration of this part, it will seem probable that the irritative effect of this morbid process may have contributed materially towards the development of the malignant disease (to which he may have had a constitutional predisposition,) at a much earlier period of life than if such an exciting cause had not been present. And, after it was produced in one part, that a similar malignant action should have exhibited itself in other organs, such as the kidneys, not immediately connected with its original seat, will excite no surprise in the minds of those acquainted with the progress of such affections.

It might have been supposed at first sight, from the boy's age, strumous complexion, emaciated habit, and tumid belly, that his disease was marasmus or mesenteric tabes (as it has been called). But some of its features at once distinguished it from that affection.

In ordinary obstruction and disease of the mesenteric absorbent vessels and glands in children, the swelling of the belly is a hard, tense, uniform, elastic enlargement, in which fluctuation is scarcely ever to be felt, or is, at any rate, from the scantiness of the fluid effused, when effused at all, exceedingly obscure and doubtful; the legs and thighs also are scarcely ever swelled. The very character of the swelling, besides, prevents the practitioner, in such cases, from feeling the enlarged mesenteric glands. There is, moreover, in mesenteric tabes no tenderness on pressure, and very little pain.

On the contrary, in this case, there was an abundance of fluid fluctuating freely in the abdomen; the cellular tissue of the legs and thighs was also much charged with serum. The shape and size of the circumscribed tumor within the abdomen could be felt, even before the quantity of the accumulated serum had been diminished by medicine, and afterwards its extent and dimensions were clearly ascertained; and, lastly, there was no tension, except that which depended on the dropsical effusion, and which disappeared with its subsidence; and no rigidity of the parietes of the abdomen, although there was very considerable tenderness on pressure, even to the last, but without fever, or any proper inflammatory symptoms.

### Case II. *Carcinoma of the Stomach.*

Alexander Forbes, aged forty-eight, machine maker, admitted into St. George's Hospital, under Dr. Chambers, on the 28th of November: complains of tormina, tenesmus, and the frequent discharge of small liquid motions mixed with blood, and says he has no proper feculent evacuations. His abdomen, which is not tense, is very tender, particularly in the region of the ascending and transverse colon. No sickness or nausea.

Pulse 100, small; skin warm; tongue white, moist; urine not passed freely, but natural in appearance; no appetite; he is much emaciated.

He says he has been ill only three weeks with the present symptoms, which are daily increasing in violence, but he has taken no medicines. He attributes his complaints to cold.

Hydrarg. Submur. gr. iij.; Opii puri gr. ss. ter die.—Milk diet.

Under this treatment, to which were afterwards added twenty-five drops of laudanum, an opiate injection every night, and mustard poultices to the abdomen, he became daily more comfortable as to his bowels, but his debility and emaciation evidently increased rapidly; the tenderness of the abdomen was distressing to the last, but no tumor or hardness was felt in any part of the belly. It may be remarked, however, that he would not allow sufficient pressure to be made on the abdomen for a satisfactory examination with reference to this point. His mouth was not made sore by the mercury. Latterly he could take no nourishment except small quantities of arrow-root and jelly, with wine or brandy; and he died on the 11th of December.

*Sectio cadaveris,* December 12th.—The stomach had an opaque white appearance externally, which was not natural. Within it a large ulcer, of the true carcinomatous character, occupied more than half the internal superficies of the organ. It extended from the cardiac close to the opening of the œsophagus, along the lesser arch of the stomach to the pylorus, two-thirds of the annular edge of which aperture were involved in the ulceration, the anterior third part only of the ring being healthy. The anterior and upper portion of the stomach was free from disease internally, except that the villous coat was somewhat thicker than natural. The pyloric extremity of the pancreas was enlarged and indurated and the induration had the character of scirrhus. There was no disease of the absorbent glands in the neighbourhood of the stomach and pancreas. The mucous lining of the ascending and transverse colon exhibited traces of small superficial ulcers which had evidently healed some time; the mucous membrane of the bowels throughout was paler and thicker than natural.

The liver was large and firm, but of a healthy colour, and not diseased in structure.

There was no disease in the chest.

The above case, which is very like those which formed the subject of discussion at a recent meeting of the Medical and Chirurgical Society, exhibits another instance of the imperfection of our present system of symptomatic nosology. In this case there was no symptom wanting of disease in the bowels, and scarcely any symptom present of disease of the stomach, not even nausea, and yet the former were found nearly healthy, whilst the latter was extensively disorganised by malignant ulceration.

From the patient's own account of his complaint, it would follow that it was only of five weeks' standing. It was obvious, however, from the appearances after death, that it must have commenced at a much earlier period. He became aware of the complaint, as it would appear, only when it interfered materially with the digestion of his food, which was then passed on from the stomach into the bowels in such a state as to produce the dysenteric symptoms which induced him to apply for relief.

Case III. *Malignant Tumor within the Thorax.*

On Thursday, 15th November, Dr. Seymour was requested to visit Mr. A. B., æt. fifty-one, who had generally enjoyed a remarkably good state of health. In May last he was attacked with cough and difficulty of breathing, which he attributed to exposure to draughts of cold air: he brought up at this time a little blood by coughing. The ordinary remedies appeared to relieve, if not entirely subdue, the symptoms of the disease, and he was again enabled to take his usual exercise, which was uncommonly violent —walking considerable distances, or riding a young and unruly horse. About six weeks ago the symptoms returned, and he coughed up a small quantity of dark-coloured blood.

At present he complains of difficulty of breathing, accompanied with constant, and occasionally violent, fits of coughing; expectoration very scanty; his inspiration is difficult, and his attempts to take a long breath are accompanied with a wheezing noise on the right side of his chest, although he fills the lungs on that side completely with air. The ribs on the left side appear fixed, and the left lung impervious to air. Pulse ninety, not weak; skin cool; lies with equal facility on either side or on his back; sleeps ill habitually, and this inconvenience has been lately much increased; tongue clean and moist; no accessions of hectic fever; complains of weight and tightness at the scrobiculus cordis, and want of appetite; bowels regular.

Capiat Pulv. Ipecac. ℈j. pro emetico statim.

November 16th.—The emetic relieved the sense of weight and tightness in the epigastric region. Pulse 100, not weak; expectoration frothy; respiration in the right side of the thorax attended with a peculiar harsh noise, like the blowing of bellows; on the left side the sound is perfectly dull, and the ribs are not raised on inspiration; countenance extremely pale and anxious; no unusual pulsation to be found or pain experienced in any part of the chest; bowels not open.

Fiat V.S. ad ℥xvj.—℞. Hydrarg. Submur. gr. iv.; Pulv. Jacobi Vir. gr. iv. M. fiat pulvis h. s. sumend.—Haust. Sennæ c. m.

17th.—Blood drawn yesterday much buffed and cupped; sonorous respiration in right side of the lungs continues; he raises slightly the ribs on the left side during respiration; pulse ninety-six, not weak.

Repet[r] V.S. ad ℥xvj.—℞. Pil. Hydrarg. gr. iij.; Scillæ recentis gr. ij. M. fiat pilula bis in die sumend.—R. T. Camphoræ c. ʒj.; Mist. Amygd. ʒx.; Muc. Gummi Acac. ʒij.; Syrupi ʒj. M. fiat haustus h. s. sumend.

17th.—Blood drawn not buffed or cupped; respiration easier, but very sonorous at intervals; pulse ninety; expectoration free, frothy.

C. C. lateri sinistr. ad ℥x. postea admoveatur Emplast. Cantharidis pectori.—Contin. alia.

18th.—The symptoms the same as yesterday. The remarkable imperviousness to the admission of air in the left lung, the absence of hectic fever, the expectoration being frothy and without the least trace of puriform matter, and the absence of pain, induced me to believe the symptoms to arise from the pressure either of an aneurismal tumor, or one formed by enlarged glands at the origin of the bronchi, especially on the left side. In consequence of this view of the case, (says Dr. Seymour,) the family were informed of my fears and my despair of its favorable termination.

The event justified the prognosis given by Dr. Seymour, as his patient gradually got worse, and died on the 2d of December.

*Examination of the body, thirty-two hours after death.*—A tumor was found in front of the chest, above the heart, situated for the most part anterior to the roots of the lungs, but surrounding the lower part of the trachea and both bronchi, the trunk and branches of the pulmonary artery, the pulmonary veins of the left side, the arch of the aorta and left carotid and subclavian arteries, but leaving the anterior part of the ascending aorta and arteria innominata uncovered. Behind, it was in contact with the œsophagus; and below, with the upper part and left side of the pericardium; thence it extended into the left lung, so that nearly half of that viscus was occupied with a similar congeries of globular tubercles, the largest about two inches and a half in diameter. The tumor of the lung adhered to the left side of the pericardium, to the diaphragm, and to the vertebræ and heads of the ribs behind, so that the lung could not be removed without tearing through the tumor. Most of the masses composing the tumor were of a white colour, but some were black in the centre, and others had begun to become soft and red in the inside. Where the tumor was in contact with the diaphragm, that membrane had become softer. A rupture had taken place, by which an effusion of blood had occurred, from the centre of one of the tubercles behind the vena cava descendens into the cavity of the pericardium, which contained about a pint of fluid blood.

The left bronchus and its branches were much lessened in diameter by thickening and pressure, and the remaining part of the lung of the left side scarcely crepitated, being filled with mucus and watery exhalation. The tumor had grown most in the lower lobe, so that very little of the texture remained; which, however, was solid. The pleura covering this lobe was much thickened, and adhered to the ribs. Many irregular white masses of condensed cellular membrane extended from the tumor into the outer part of the lung, thicker and less ligamentous than the bands usually are in cancerous tumors.

The disease did not reach beyond the root of the right lung, which was red and full of fluid, and the pleura contained a number of white spots of a cartilaginous consistence, which were of the size of a split pea.

A tumor similar to that in the chest, and about the size of a small orange, was situated above the head of the pancreas.

The rest of the viscera were healthy.

This case affords a well-marked example of a disease, which, although it is known to practitioners of long and extensive experience, is nevertheless rare.

It appears to have been known to Morgagni, who has described it as a peculiar appearance, as resembling fatty tumors, *(quasi steatomata,)* in Art. 22, Epist. 22. It has been much commented on by modern French writers. Bayle terms it cancerous tubercle of the lungs; and by Laennec it is called *Encéphaloides des poumons*, because, in its advanced or third stage, it occasionally resembles in appearance and consistence medullary matter. It is the Fungous Hæmatodes of English writers. Bayle has the merit, among the French, of having first described it under the title *Phthisie cancereuse.*

Until the deviation from the laws which govern the animal body in a state of health, which precedes these formations, be understood, we must be satisfied with palliative treatment.

By relieving the symptoms which arise from pressure, by moderate venesection, by enjoining perfect rest and strict diet, restraining the irritating cough by mild opiates, and supporting the occasionally fainting powers with diffusible stimulants, we shall contribute to the comfort of the patient, and delay the fatal event.

